# RhizoTubes as a new tool for high throughput imaging of plant root development and architecture: test, comparison with pot grown plants and validation

**DOI:** 10.1186/s13007-016-0131-9

**Published:** 2016-06-07

**Authors:** Christian Jeudy, Marielle Adrian, Christophe Baussard, Céline Bernard, Eric Bernaud, Virginie Bourion, Hughes Busset, Llorenç Cabrera-Bosquet, Frédéric Cointault, Simeng Han, Mickael Lamboeuf, Delphine Moreau, Barbara Pivato, Marion Prudent, Sophie Trouvelot, Hoai Nam Truong, Vanessa Vernoud, Anne-Sophie Voisin, Daniel Wipf, Christophe Salon

**Affiliations:** UMR 1347 Agroécologie AgroSup/INRA/uB, 17 Rue Sully, BP 86510, 21065 Dijon Cedex, France; Inoviaflow, 210 avenue de Verdun, 39100 Dole, France; INRA, UMR759 LEPSE, 2 Place Pierre Viala, 34060 Montpellier, France

**Keywords:** High-throughput, Growth, Drought, Nitrogen availability, Plant–microorganism interactions, Image acquisition, Phenotyping, Plant roots, RhizoCab, RhizoTubes

## Abstract

**Background:**

In order to maintain high yields while saving water and preserving non-renewable resources and thus limiting the use of chemical fertilizer, it is crucial to select plants with more efficient root systems. This could be achieved through an optimization of both root architecture and root uptake ability and/or through the improvement of positive plant interactions with microorganisms in the rhizosphere. The development of devices suitable for high-throughput phenotyping of root structures remains a major bottleneck.

**Results:**

Rhizotrons suitable for plant growth in controlled conditions and non-invasive image acquisition of plant shoot and root systems (RhizoTubes) are described. These RhizoTubes allow growing one to six plants simultaneously, having a maximum height of 1.1 m, up to 8 weeks, depending on plant species. Both shoot and root compartment can be imaged automatically and non-destructively throughout the experiment thanks to an imaging cabin (RhizoCab). RhizoCab contains robots and imaging equipment for obtaining high-resolution pictures of plant roots. Using this versatile experimental setup, we illustrate how some morphometric root traits can be determined for various species including model (*Medicago truncatula*), crops (*Pisum sativum, Brassica napus, Vitis vinifera, Triticum aestivum*) and weed (*Vulpia myuros*) species grown under non-limiting conditions or submitted to various abiotic and biotic constraints. The measurement of the root phenotypic traits using this system was compared to that obtained using “classic” growth conditions in pots.

**Conclusions:**

This integrated system, to include 1200 Rhizotubes, will allow high-throughput phenotyping of plant shoots and roots under various abiotic and biotic environmental conditions. Our system allows an easy visualization or extraction of roots and measurement of root traits for high-throughput or kinetic analyses. The utility of this system for studying root system architecture will greatly facilitate the identification of genetic and environmental determinants of key root traits involved in crop responses to stresses, including interactions with soil microorganisms.

## Background

Agriculture now faces a dual challenge, feeding an ever-growing population estimated to double by 2050 [1] while preserving terrestrial resources and the quality of the environment. In the context of climate change, agriculture appears to be both causal and exposed to detrimental environmental impacts. Thus, there is an urgent need to improve and/or stabilize the productivity of crops under stressful climate conditions, reducing chemical fertilizer use and optimising water availability. This could be achieved by exploiting genetic diversity in the design of new plant varieties, which use soil resources more efficiently. Specifically, at the plant–soil interface, it is necessary to target crop plants with better developed and/or more efficient root systems for assimilating soil resources. The benefits of symbiotic plant–microbe associations also merit exploration.

For some years now, both genetics and associated breeding tools, and sequencing resources, have increased exponentially while becoming cheaper. However, the precise characterization of gene expression in a variety of environments (i.e. phenotyping) at high-throughput has become the main bottleneck in plant breeding [2, 3]. It is thus mandatory to develop tools and algorithms for quantifying structural traits of complex/varied shoot/root systems at both high-throughput and resolution in order to reconcile them with the huge amounts and fast flow of genomic data.

Most phenotyping efforts have mainly focused on shoot traits such as leaf area, biomass, grain yield and product quality [4–6]. However, despite the key roles of the root system for the plant functioning (water and nutrient acquisition, mechanical support, interaction with soil microorganisms…), plant breeders generally shy away from selection in the field for root traits as their characterisation requires an important investment in terms of time and manpower [7, 8].

Technical difficulties in accessing roots non-destructively and nonetheless dynamically within the soil for phenotyping root traits are an obvious explanation for the scarce attention given to root phenotyping. Moreover, the root system is very plastic in response to both abiotic and biotic environmental factors. Environmental factors known to influence Root System Architecture (RSA) are (1) abiotic factors such as temperature [9], water stress and soil structure [10–13], soil nutrient availability including nitrogen (N), phosphorus, iron and sulphate [14, 15] and (2) biotic factors among which are soil microorganisms [16].

Root systems may be characterized not only in natural field soil [13, 17, 18] but also in hydroponics [19–21], gellan gum, gel chambers and agar plates [9, 22–25]. Soil structure is artificially modified in pots, although some set-ups are able to mimic soil compaction [9, 11, 24]. Root phenotyping in growth pouches is consistent with results acquired in pots and may permit the study of genetic determinism of plant traits and in the selection of genotypes contrasted for root and nodule features [26].

Root system enclosures comprise soil-filled tubes or rhizotrons [9, 24, 27, 28], which allow non-invasive dynamic measurements of RSA through a transparent exterior screen. Root system enclosures allow either 2D [20, 24, 27, 28] or 3D visualization [22, 29, 30]. For example, 2D root observation is provided by growth pouches and plants grown between paper in rhizoslides [26, 31–33], while 3D observation is possible in solid gel matrices such as agar or gellan gum [22, 29, 34, 35] or hydroponics [20] or transparent soil [36]. Roots may also be phenotyped when cultivated in the field [17, 37].

So far, most of the techniques developed for high throughput RSA phenotyping involve the use of young seedlings, which are not always representative of mature plants [38, 39], even though seedling root phenotype may be a good predictor of later developmental stage morphometry [7]. However, it remains crucial to phenotype older and thus more developed root systems because for most plants root number can increase up to late stages [14, 26, 40] but also because the different root types (e.g. lateral roots and adventitious roots) of various eudicots and monocots that develop throughout plant growth [41] evolve in their efficiency for resource capture and nutrient acquisition [42].

Image-based methods (e.g. relying on the use of scanners or cameras) are mostly used for measuring the size, architecture, and other structural shoot and root traits at high throughput. These methods allow hundreds of plants to be phenotyped daily given the short time required for image acquisition [9, 20, 25, 32, 33, 43, 44]. Other methods such as 2D neutron radiography and tomography [45, 46] are only low-throughput. 3D-imaging methods allowing in situ visualization of roots in soil are based on X-ray computed tomography [47, 48] or nuclear magnetic resonance imaging [49, 50].

The aim of our study was to test the suitability of our tools for growth and phenotyping of different plant species addressing different research questions. These tools comprise RhizoTubes (1200 in total), which are cylindrical rhizotrons that allow full 2D visualization of the root system of a single or up to six plants simultaneously, for a maximum plant shoot height of 1 m and around 6–8 weeks age depending on both environmental conditions and plant species. The RhizoCab is designed to take images of the entire root systems of plants growing in RhizoTubes, and also permits a focus on some parts of the root systems. The main objectives of this paper were: (1) to compare growth of various plant species in RhizoTubes with that in pots; (2) to assess if root images could be acquired by the RhizoCab and their quality for further phenotyping of traits; (3) to compare the values of the phenotypic traits measured manually for plants growing in RhizoTubes with those of plants in pots, in response to modification of their biotic or abiotic root environment.

As such, the suitability of the methods presented here was tested in a number of species by comparing root traits measured in RhizoTubes with those acquired from “classic” growth in pots under similar environmental conditions. Among crop species studied here, oilseed rape and legumes are very important crops in European cropping systems. Oilseed rape is phylogenetically close to *Arabidopsis thaliana* but has a much larger root system, which remains poorly characterized. Legumes possess unique features compared to other plants due to their symbiosis with N-fixing soil bacteria. For both non-legume and legume species, nitrogen availability in soil is known to induce major changes in shoot and root biomass and architecture. Grapevine plantlets in pots are generally obtained from herbaceous cuttings and the suitability of RhizoTubes was tested for this growth possibility.

Thus, we investigated (1) the use of RhizoTubes for growing grapevine and its mycorrhization, (2) the nodulation ability of the root system of legume plant species *Pisum sativum* and *Medicago truncatula* in response to nitrogen and water availability, (3) the contrasting responses of oilseed rape and *Vulpia myuros* to nitrogen availability, and (4) the ability of bacterial strains to persist on pea and wheat roots.

## Methods

### Automated phenotyping of root system architecture and shoot growth

The Plant Phenotyping Platform for Plant and Micro-organism Interactions (4PMI) is hosted by the UMR Agroécologie (INRA Dijon, France, https://www6.dijon.inra.fr/umragroecologie/Plateformes/Serres-PPHD). 4PMI is an automated phenotyping platform based on conveyors (LemnaTec, Würselen, Germany). It is composed of four different greenhouses where environmental conditions can be varied independently (temperature, light, hygrometry, individual plant watering regime). For each greenhouse, conveying lanes (in total 60 lanes for 4PMI) carrying 26 carts each (in total 1560 pots), are used to transport plants either towards two watering units or to the imaging units. Watering units consist of two weighing terminals (ST-Ex, Bizerba, Balingen, Germany) and high-precision pump-watering stations (520Du, Watson Marlow, Wilmington, MA, USA). The visible imaging unit designed to acquire non-invasive shoot images of either pots or RhizoTubes is composed of a 3D image acquisition cabin with top and side cameras (Basler piA2400-17gm/gc with a motorized lens Pentax C-Mount 12.5–75 mm C6Z1218M3 2/3″ 6× Megapixel, Basler AG, Ahrensburg Germany) and illumination (HE 28W/865, OSRAM, Augsburg, Germany). The top camera can take zenithal images while the side camera mounted at an angle of 90° to the vertical axis of the plant allows acquiring shoot images at different angles of rotation, whose number and amplitude depends upon the plant type. Circulation of plants via conveyors, image acquisition and watering is regulated by a control personal computer using the LemnaLauncher software bundle (LemnaTec, GmbH, Würselen, Germany).

#### RhizoTube description

**Fig. 1:**
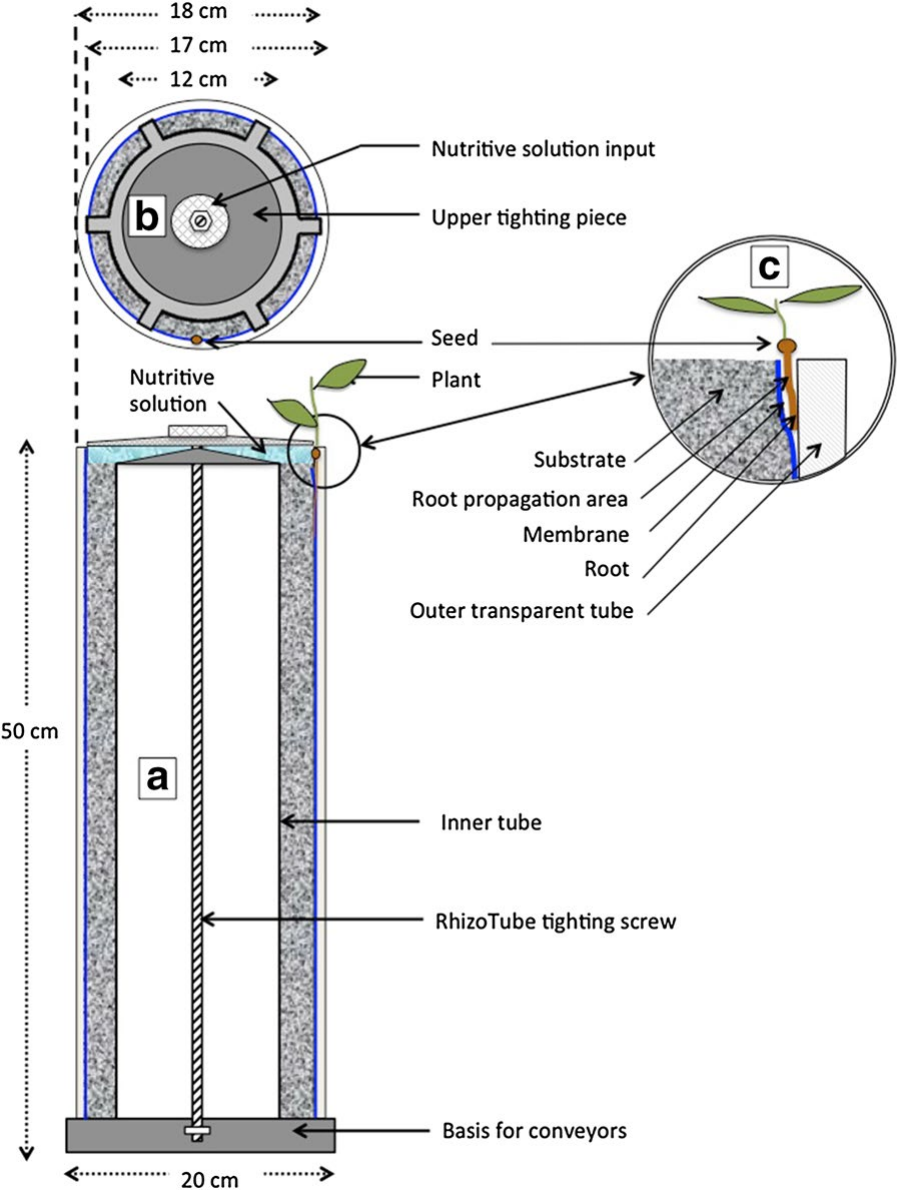
The RhizoTube (**a**) is composed of concentrical tubes (an outer transparent PMA tube, an inner inox tubes) tighted together to the bottom and upper parts of RhizoTubes thanks to an axe, a bottom bolt and an upper star shaped tighting piece (**b**). Nutri solution supplied by the top (**b**) of the RhizoTube flows within the RhizoTube to the substrate, filled in between the inner tube and a membrane, permeable to nutrients, water and microbes but not to plant roots. This membrane has been tinted in blue with physiological inert ink to avoid any interference with plant growth. The seeds are placed at the top of the RhizoTube (**c**) and the plant root grows in its root propagation area (**c**) defined as the space between the outer transparent tube and the membrane. RhizoTubes are installed on conveyors thanks to a special adapted basis, which contains a unique RFID per Rhizotubes

RhizoTubes and RhizoCab were designed in close collaboration with Inoviaflow (Dole, France). RhizoTubes are cylindrical rhizotrons where plants grow and can be phenotyped dynamically. They are 18 cm in diameter and 50 cm high, and weigh approximately 12 kg. Because our visible imaging unit is about 1.6 m, this allows working with plant shoots 1.1 m high, which is sufficient for the time span of our plant root observations on the various species we work on. RhizoTubes are composed (Fig. 1) of concentric tubes, which delimit the outside to the inside of RhizoTube the root growing zone from the substrate zone and lastly the center where nutrient solution is supplied. The root growing zone lies between an inner permeable membrane (mesh size of 18 µm) and the external outer transparent polymethylmethacrylate tube (Fig. 1), separating the plant root from the soil. This membrane has been designed to be permeable to nutrients, water, plant rhizodeposits and microorganisms but it does not allow roots to pass through. As such the full root system is confined in two dimensions (Fig. 1c) and can be photographed using the Rhizocab camera through the outer transparent tube. A central inner tube defines the substrate thickness (about 2.5 cm, around 2.5 L of substrate) (Fig. 1). From one up to six plants can be sown simultaneously in a RhizoTube. Nutrient solution is supplied at the top of the RhizoTube and flows to the surrounding substrate zone.

**Fig. 2:**
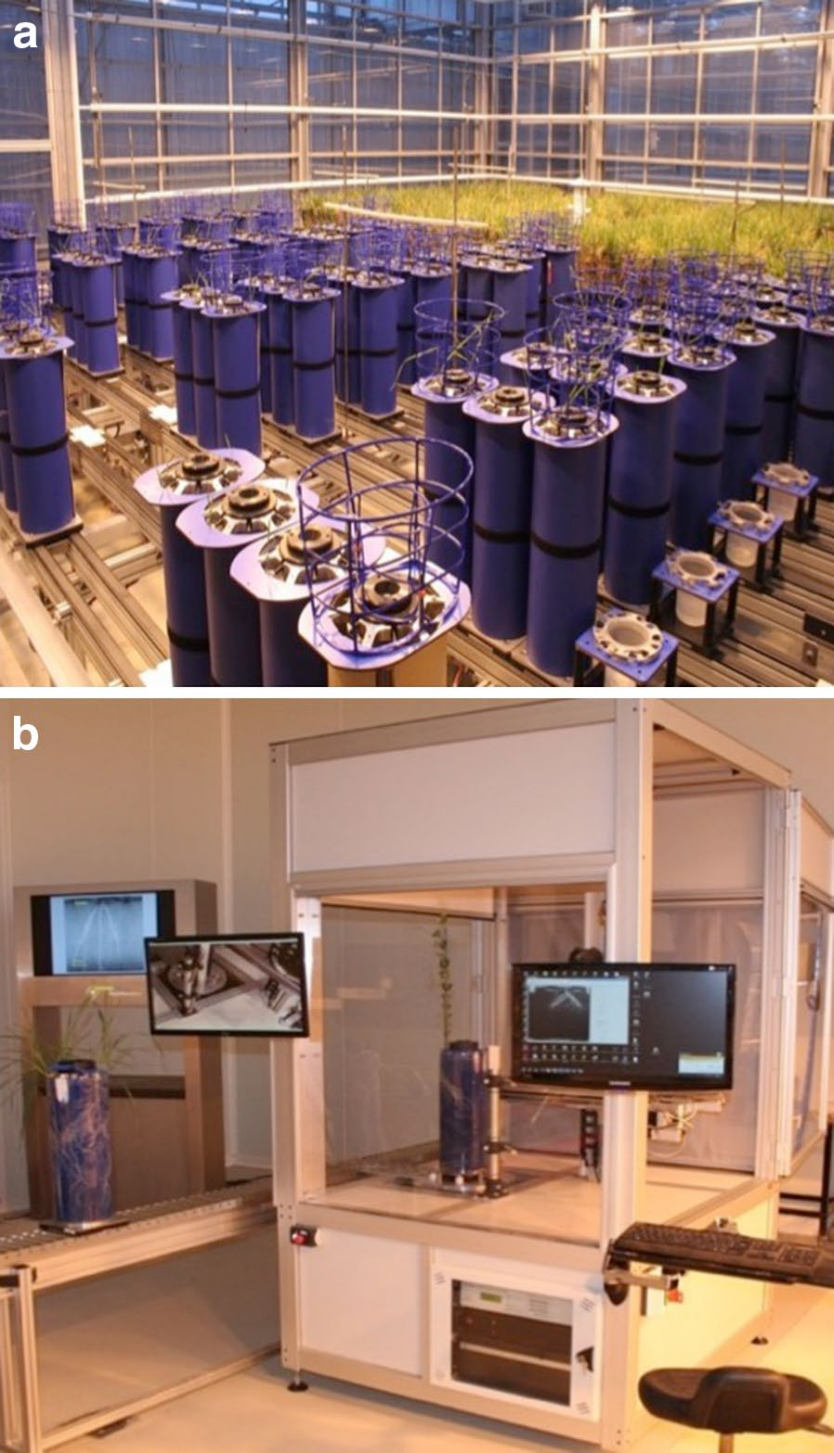
View of **a** a greenhouse with both RhizoTubes and pots on conveyors and **b** the root phenotyping RhizoCab. The RhizoCab has a brushless motor turntable which allows the RhizoTube to turn while image acquisition is synchronized. RhizoTubes are installed on conveyors (**a**) where they are automatically moved to solution stations where fertirrigation is gravimetrically controlled. The operator can bring the RhizoTube to the RhizoCab (**b**). An operator can define all of the desired parameters (light wavelength, image resolution, file name etc.)

Conveyors (Fig. 2a) transport either pots or RhizoTubes to the high-throughput sequential phenotyping cabins to phenotype shoots at various wavelengths. Each of the growing units (RhizoTube or pot) is uniquely identified by a Radio Frequency IDentification (RFID) chip in its base. As for pots, when needed, RhizoTubes are conveyed either to the watering stations where they can receive the target amount of nutrient solution, with a possibility of two different ones. Each RhizoTube is drained to evacuate excess nutrient solution through a hole in its base. The solution is automatically released from RhizoTubes when they pass through a drainage station. An opaque shell to shade roots from light covers the transparent outer tube of the RhizoTubes. This shell avoids algal growth in the soil using two half-shells of aluminium, which also provides insulation. This opaque shell is automatically and mechanically opened and smoothly lifted by about 5 mm above the RhizoTube basis by an arm equipped with a plier when RhizoTubes enter the RhizoCab. Opening the shell by about 2 cm allows the light to illuminate only the zone where roots can be imaged by the RhizoCab camera. Lifting the shell permits the RhizoTube to rotate on the RhizoCab turntable.

For some plant species (e.g. wheat), seeds are germinated on moistened filter paper and then installed within the RhizoTube. For others (e.g. pea) seeds can be directly installed within the RhizoTube (Fig. 1c). In the case of grapevine, herbaceous cuttings were prepared as previously described [51, 52] until they developed 6 leaves, and then transplanted in the RhizoTube.

#### Acquisition of root images with RhizoCab

The RhizoCab (Fig. 2b) is an aluminium and glass box (1.5 m width, 1.5 m deep and 2.5 m high). RhizoTubes have to be loaded in the RhizoCab with a trailer equipped with rolling tubes to facilitate handling the RhizoTubes. Within the RhizoCab, three special LED lamps (465, 525, 625 nm wave length) are synchronized with image acquisition process using a high definition camera (Zeiss 50 mm f/2 Makro-Planar lens and Basler raL12288-8gm camera) mounted at an angle of 90° to the vertical axis of the RhizoTube. This camera can be moved in 2D according to the operator’s needs. The RhizoCab software manages the root phenotyping process: while the RhizoTube is rotated on the turntable by a brushless motor on its main axis, the camera simultaneously takes a 1 pixel wide × 12000 pixels high image for the total RhizoTube height. For a given wavelength, an image of the complete outer area of the RhizoTube is acquired in 10 s. If needed, the operator can zoom on a desired area of the root system for high-resolution images. For each resolution position, the camera was calibrated to allow retrieval of real size dimensions from pixels. The calibration process considers that all exterior tubes are perfectly similar. The three wavelengths of the LEDs are used to reproduce a color image in the RGB (red, green and blue) colorimetric model. The monochrome camera is then able to reproduce the light intensity corresponding to each selected illumination. This method corresponds to the use of a color camera plus white light, but provides an image spatial resolution four times greater. Because there is no need to use either a color filter before the sensor or pixels interpolation from the sensor to retrieve an image, 100 % of the acquired pixels are clean and useful. For any resolution, the final definition of the color image is 12,000 × 12,000 pixels with a file size of 411 MB. When the RhizoCab is implemented in one of the 4PMI phenotyping cabins, by conveying up to 120 RhizoTubes can be phenotyped per hour. The RhizoCab is the prototype of a high throughput root phenotyping cabin (RhizoCabHT), which has been implemented in one of the sequential phenotyping cabins of 4PMI in BMP format. For storing images, the most suitable format such as the commonly used PNG format does not lead to loss of information by compression. While RhizoTubes are manually loaded in the RhizoCab, which limits the phenotyping throughput to about 100 RhizoTube per day, RhizoTubes are automatically conveyed, like pots are on 4PMI, inside RhizoCabHT so that the throughput of 4PMI will allow root imaging of our 1200 RhizoCabHT within a day.

### Experiments performed

A series of experiments were conducted to assess (1) how the RhizoTube affects plant cultivation as compared to growth in pots and (2) the quality of images captured by RhizoCab for further phenotyping. Plant species and environmental conditions were chosen so that to (1) represent various research thematics of our unit, (2) assess the suitability of our system for different plant root architectures, and (3) taking into account interactions with microorganisms leading to specialized structures such as nodules (e.g. legume plants) or mycorrhiza (grapevine). Experiment 1 was performed on grapevine (*Vitis vinifera* L.) to assess survival within RhizoTubes of herbaceous cuttings, the usual means of propagation. Experiments 2 and 3 were conducted to characterize root architecture of nodulated pea roots (*Pisum sativum*) when grown in RhizoTubes or in pots and the influence of either different genotypes or varying soil nitrogen availability. In experiment 4, the responses to soil mineral N availability of a crop (*Brassica napus*) and a weed (*Vulpia myuros*) species were compared between RhizoTubes and pots. In experiment 5 the impact of water deficit on legume plants (pea and *Medicago truncatula*) was compared in pots and RhizoTubes. As RhizoTubes were developed to provide a powerful tool for investigating plant–microorganism interactions, experiment 6 aimed at establishing the persistence of microorganisms on roots within the RhizoTube rooting medium following their early inoculation within the RhizoTube rooting medium.

#### Growing conditions and plant material

A summary of the experiments (Table 1) depicts the environmental setup. All of the experiments were conducted in a greenhouse so that environmental conditions including the substrate used for growing plants and climatic conditions (light, temperature and hygrometry) were similar whether plants grew in pots or in RhizoTubes (Table 1). In experiment 1 *Vitis vinifera* L. cv. Marselan (Cabernet sauvignon × Grenache) obtained from herbaceous cuttings was grown in RhizoTubes and mycorrhized with Symbivit^®^Pro (Inoculum plus, Dijon, France). In experiments 2 and 3, pea plants (cv Kayanne in experiment 2, cv Cameor and Kayanne in experiment 3) were cultivated with 10 mM N (experiment 2) or without nitrogen in the form of nitrate in the nutrient solution (experiment 2, 3 and 5). In these experiments, plants were well watered (WW) to maintain soil water content between 85 and 100 % (w/v) in pots and RhizoTubes. Roots of plants in RhizoTubes grow between the outer transparent tube, which has no water retention capacity, and the membrane allowing nutrient solution to flow freely from the substrate to the roots. As such, roots of plants growing in RhizoTube have a “limited” access to water as compared to those of plants growing in pots and a contrasted growth. While in preliminary experiments plants in RhizoTube and pots supplied with both similar amounts and frequency of nutrient solution did not lead to similar growth conditions between containers, these were reached thereafter by increasing the frequency and amount of nutrient solution to plants in RhizoTubes as compared to plants in pots. To avoid root flooding and adjust plant growth between containers RhizoTubes were equipped with a drainage hole and conveyors with an automatic flushing station. In experiment 5, for water-stressed (WS) *Medicago* (A17 genotype) and pea plants grown in pots, watering was stopped 15 days after sowing until soil water had decreased to 40 % (w/v) at which level it was maintained for 12 days. When plants were grown in RhizoTubes they received sequential additions of lower predefined doses of nutrient solutions during the day. In experiment 4, *Brassica napus* (genotype Kadore) and *Vulpia myuros* received two nitrate concentrations in the nutrient solution either 0.625 or 10.5 mM. In experiment 6, pea (cv James) and wheat (cv Arezzo) plants were cultivated in a growth chamber.

**Table 1:** Species, containers and environmental conditions of the different experiments

Exp.	Species (*genotype*)	Experimental treatment	Number of replicates: pots (P) and RhizoTubes (RT)	Pot size (L)	Mean day temp (°C)	Mean night temp (°C)	Photoperiod (h)	RH	Nutrient solution	Inoculation	Substrate
1	Grapevine (*V. vinifera* L. cv. Marselan)	With or without mycorrhization	0 P, 4 RT	–	24	18	16	60 to 80 %	Solution 1	Symbivit^®^ Pro) (1 %v) for the “mycorrhiza” condition	Substrate A
2	Pea (*Kayanne)*	Low and high mineral nitrogen availability	5 P, 5RT	4	22	18	16		Solutions 3 and 4	Rhizobium P221 (10^8^ CFU per plant)	
3	Pea (*Kayanne)*	Genotype comparison	5 P, 5RT	4					Solution 2		
3	Pea (*Caméor)*		5 P, 5RT	2					Solution 3		Substrate B
4	*Brassica napus (Kadore)*	Low and high mineral nitrogen availability	5 P, 5RT	4					Solutions 3 and 4		Substrate A
4	*Vulpia myuros*		5 P, 5RT	4							
5	Pea (*Caméor)*	Optimum water nutrition and water deficit	5 P, 5RT	2					Solution 2	Rhizobium P221 (10^8^ CFU per plant)	Substrate B
5	Medicago truncatula (J7)		5 P, 5RT	1					Solution 3	Rhizobium MD4 strain (10^8^ CFU per plant)	
6	Wheat (*Triticum aestivum* cv. Arezzo)	Monoculture or association between pea and wheat	0P, 4 RT	–	20	20	15	90 % (first week only) then 60 %	Solution 3	*P. fluorescens* C7R12 (10^10^ CFU per plant)	Substrate A
6	Pea (*Pisum sativum* cv. James)		0P, 4 RT	–						*R. leguminosarum* P221 (10^7^ CFU per plant) and *P. fluorescens* C7R12 (10^10^ CFU per plant)	

The substrates and nutrient solutions used for plants grown in pots and RhizoTubes were similar for a given experiment. Substrate “A” was composed of a mixture of equal volumes of clay beads (Sorbix US-Special G, Damolin, Etrechy, France) and atapulgite (ARGEX NV, Belgium). Substrate “B” was composed of a mixture of equal volumes of sand (Biot sand, 0.8 to 1.6 mm, silica 100 %, Silice et réfractaires de la méditerranée, France) and perlite (Perligran Premium, Knauf Aquapanel, Germany). The nutrient solution numbered “1” used for control plants contained Plantin (10–10–10), Magplant–S and for Myc. plants Plantprod (14–0–14), NaH2PO4, 2H_2_O (1 %). The nutrient solution without mineral nitrogen numbered “2” was composed of 0.8 mM K_2_HPO_4_, 1.0 mM MgSO_4_, 2.5 mM CaCl_2_, 0.7 mM K_2_SO_4_ and 0.2 mM NaCl. The nutrient solution numbered “3” containing low amounts of mineral nitrogen (0.625 mM N) was composed of 0.16 mM KNO_3_, 0.24 mM Ca(NO_3_)2, 0.8 mM K_2_HPO_4_, 1 mM MgSO_4_, 2.27 mM CaCl_2_, 0.62 mM K_2_SO_4_ and 0.2 mM NaCl. The nutrient solution composed of high mineral nitrogen content (10 mM N) numbered “4” contained 1.88 mM KNO_3_, 2.81 mM Ca(NO_3_)_2_, 0.56 mM K_2_HPO_4_, 1 mM MgSO_4_, 2.5 mM NaNO_3_. The nutrient solutions 2 to 4 were supplemented with 50 µM iron Fe III-(EDTA), and oligo-elements. Oligo-elements were provided as 32 µM H_3_BO_3_, 10 µM MnSO_4_, 0.77 µM ZnSO_4_, 0.15 µM H_24_N_6_O_24_Mo_7_ and 0.32 µM CuSO_4_

In order to check if microbial strains could persist in RhizoTubes, the bacterial strain *Pseudomonas fluorescens* C7R12 was inoculated after sowing pea and wheat plants. The goals of this assay was to: (1) characterize the bacterial cell concentrations on plant roots after 4 weeks of culture (measure of bacterial colonies on KING B agar plates) and (2) test if the method used to disinfect RhizoTubes (sodium hypochlorite solution) was effective enough to eliminate *P. fluorescens* cells after harvesting to avoid microbial contamination of the following cultures.

The bacterial strain *Pseudomonas fluorescens* C7R12 [52] was inoculated after sowing at a concentration of 10^10^ Colony Forming Unit (CFU) per plant. During photoperiod (daylength varying from 15 to 16 h according to the experiment, Table 1), plants were continuously illuminated with a lower threshold of 300 µmol m^−2^ s^−1^ provided by supplemental illumination using 400 W lamps (HPS Plantastar, OSRAM, Munich, Germany) when incident solar radiation dropped below 300 W m^−2^.

#### Harvest and measurements

Root systems of plants grown in RhizoTubes were imaged using the RhizoCab. Plants were taken off the conveyors and transferred to a laboratory to harvest the root system for manual root phenotyping This was straightforward as RhizoTubes were dismounted using a pneumatic device to slide vertically the transparent outer tube and gain access to soil-free plant roots. For the pot experiments, roots had to be separated from the soil substrates and then gently washed to eliminate soil particles.

#### Phenotypic trait characterization

Roots issued from plants growing in pots and RhizoTubes were placed in a tray with some water to disentangle roots rapidly and manually. Root architecture phenotypic traits were determined manually. Root number and length including different root orders, spatial distribution of roots and nodules were measured for each section (experiment 3) where roots were cut every two cm.

Plants were subsequently harvested for characterization of the growth of the different compartments (biomass of shoots, roots, nodules, number of nodules…) at different periods according to the experiment and plant species. Plant leaf area was measured with a leaf area meter (LICOR 3100C, Lincoln, Nebraska, USA) together with shoot and root biomass at day 51 in experiment 4. Dry weights of both roots and shoots were determined after oven-drying samples at 80 °C for about 48 h. In experiment 1, the total mycorrhization level (frequency, F%) was measured [53]. In experiment 6, the density of *P. fluorescens* C7R12 on pea and wheat roots was checked at the end of the experiment through serial dilution and counting on KING B (Sigma-Aldrich, Saint-Quentin Fallavier, France) agar plates.

#### Statistical analysis

For each measurement, experiments were conducted with independent biological replicates consisting of one individual plant either in a pot or in a RhizoTube. Analysis of variance (ANOVA) was performed (Excel software) to compare trait values between pots and RhizoTubes and marked by an asterisk when significantly different (*, P < 0.05).

## Results

In order to verify that 2D growth in RhizoTubes allows unbiased and reproducible measurement of plant responses to abiotic or biotic factors, contrasted species and/or genotypes of *B. napus*, *V. myuros*, *P. sativum*, *M. truncatula*, *V. vinifera* and *T. aestivum* were grown both in RhizoTubes and in pots, and exposed to similar variations in either nitrate or water availability, or inoculated with soil microorganisms, according to the experiments. Shoot/root responses to these variations were measured and compared for both types of rooting container.

### Growth and mycorrhization of grapevine plants in RhizoTubes

**Fig. 3:**
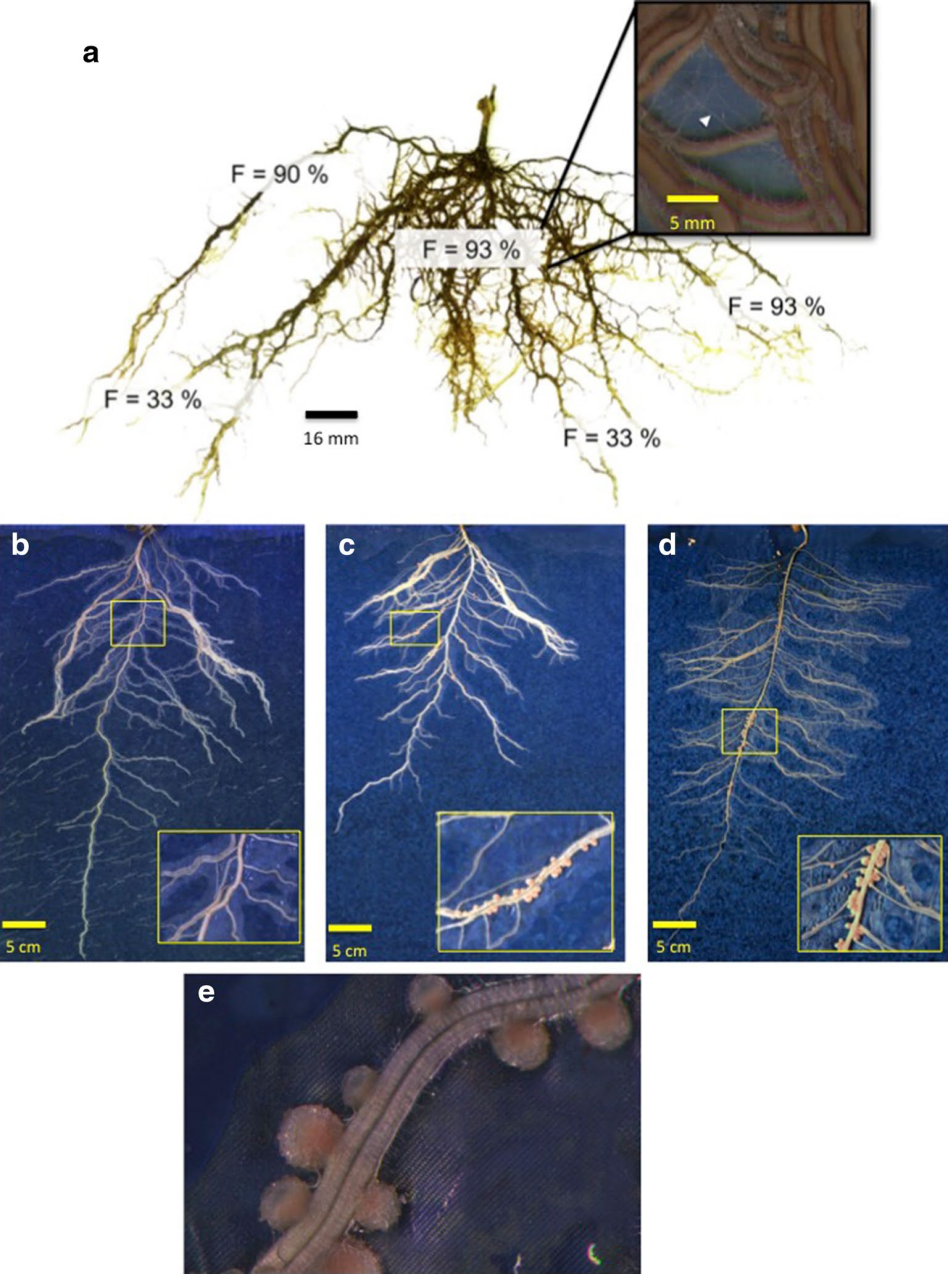
Examples of images (600) taken by RhizoCab of plant cultivated for 51 days (**a**), *Pisum sativum* plant (Cameor genotype) cultivated for 18 days with 10 meq soil mineral nitrogen (**b**) or without soil mineral nitrogen (**c**), *Pisum sativum* plant (Kayanne genotype) cultivated for 18 days without soil mineral nitrogen (**d**). Details of zone where either mycorhize can be seen or nodules (**e**) easily detected are indicated, with a resolution of 3600 (i.e. a pixel equals 7 µm)

RhizoTubes are suitable for the growth and development of grapevine plantlets obtained from herbaceous cuttings. We observed a well-balanced development of both shoots and the root system. Furthermore, no symptoms of root alteration were detected. Experiment 1 was therefore performed to check whether these systems are adapted to the production of mycorrhized grapevine plants. The shoot/root dry biomass ratio (0.57) measured in RhizoTubes was similar to the shoot/root dry biomass ratio calculated for pot-grown plantlets, indicating that overall plant development was not modified in RhizoTubes (data not shown). As observed in Fig. 3a, within RhizoTubes the number and diameter of the roots, and overall root architecture (namely extent of branching) was determined. The total mycorrhization level (frequency, F%) was around 60 % and a root age-dependent extent of mycorrhization was observed (Fig. 3a).

### Nodulated root architecture in RhizoTube compared to that in pot: the pea example

In experiment 2, pea plants (cv Kayanne) were supplied with a high level of mineral nitrogen. Under these conditions, root nodulation did not occur, neither in pots nor in RhizoTubes (Fig. 3b).

**Fig. 4:**
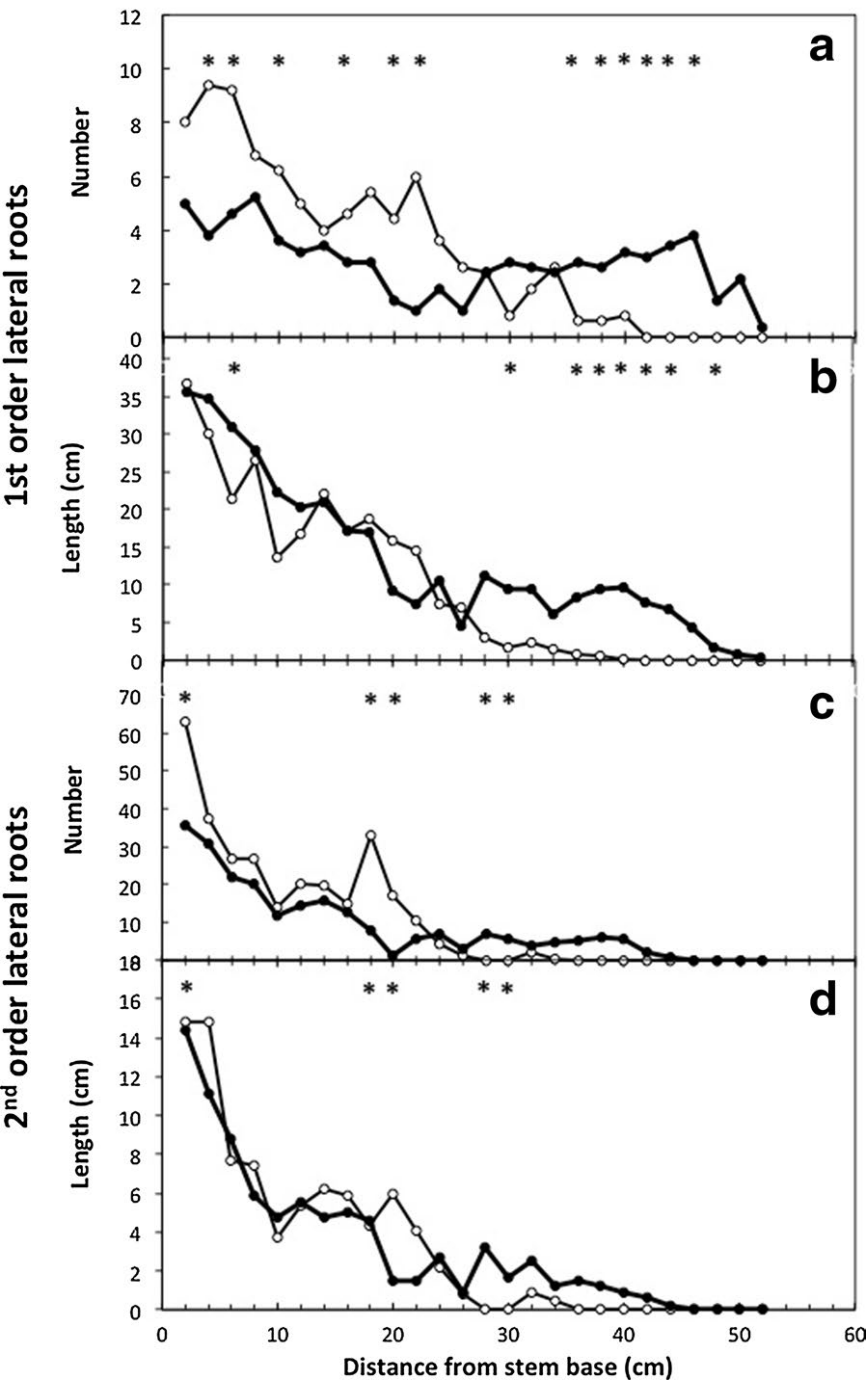
Root architecture phenotypic traits *Pisum sativum* (Kayanne genotype) grown for 28 days in pots (*open circle*) or RhizoTubes (*closed circles*) with 10 meq soil nitrogen: number of lateral root (**a**); root (**b**); number of secondary roots (**c**); length of longest secondary roots (**d**). Data are given as the mean ± SE (n = 4). *Asterisks* indicate significant differences between traits measured in RhizoTube or in pot for P < 0.05

Both total plant dry matter and root dry matter were similar in pots or RhizoTubes 28 days after sowing (Table 2). Root phenotype analysis yielded descriptors such as the length of main root (Table 2), and architectural traits such as the distribution along main root of the density of the first- (Fig. 4a) and second- (Fig. 4c) order lateral roots and of their maximal length (Fig. 4b, d). The length of the main root was 47 % higher in RhizoTubes compared to pots (Table 2). The root distribution profiles along the main root showed that number and maximal length of first order lateral root roughly evenly decreased with depth in pots (Fig. 4a, b). In RhizoTubes, they also decreased from main root base, similarly to the situation observed in pots 25 cm more below from the hypocotyl (Fig. 4a, b). There were more first order lateral roots in pots than in RhizoTubes from the hypocotyl down to 25 cm below. More than 25 cm below the hypocotyl, this was inversed: while both the number of first order lateral roots and their maximum length reached a plateau in RhizoTubes, in pots they decreased down to zero (Fig. 4a, b). Conversely, the number and maximum length of second-order lateral roots were similar throughout the distribution profile for plants growing in pots and in RhizoTubes (Fig. 4c, d).

**Table 2:** Plant and root biomass, main roots length of pea genotype in pots or RhizoTubes

	Total plant dry matter (g/plant)	Root dry matter (g/plant)	Main root length (cm)
Pots	2.04 ± 0.34 (A)	0.28 ± 0.04 (A)	33.8 ± 8.7 (A)
RhizoTubes	1.93 ± 0.33 (A)	0.35 ± 0.06 (A)	49.6 ± 0.05 (B)

Total plant (i.e. shoots and roots) dry matter, length of main roots of Kayanne genotype (*Pisum sativum*) cultivated in pots or RhizoTubes with 10 mMeq soil mineral nitrogen were measured after 28 days after sowing. Letters in parentheses indicate significant differences (P < 0.05) between traits measured in RhizoTube or in pot

As quoted previously for Kayanne, addition of nitrate prevented nodulation in both rooting media (Fig. 3b). In experiment 3, two pea genotypes (cv Caméor and Kayanne) were grown without mineral N and inoculated with symbiotic rhizobial strains. Root images acquired using the RhizoCab showed that nodulation occurred both in pots and RhizoTubes on the main and lateral roots when nutrient solution was depleted of mineral nitrogen (Fig. 3c, d).

When grown for 18 days in the absence of soil mineral N, Cameor and Kayanne pea genotypes displayed similar total plant biomasses but had slightly contrasted shoot/root biomass ratios. Kayanne invested much less biomass in its nodulated roots than in its shoots compared to Cameor, (Table 3). In pots, Kayanne nodules were half the mass of those of Cameor as shown by their respective mean nodule weight (Table 3). When plants were grown in RhizoTubes, similar values to those found for plants growing in pots were obtained for total plant biomass, and mean nodule weight while the ratio of shoot to root biomass was slightly lower (Table 3).

**Table 3:** Phenotypic traits of two pea genotypes in RhizoTubes or pots

	Plant dry matter (g/plant)	Shoot/root dry matter	Mean nodule dry matter (mg/nodule)
Caméor			
Pot	0.43 ± 0.04 (A)	1.478 ± 0.201 (A)	0.222 ± 0.095 (A)
RhizoTube	0.39 ± 0.03 (A)	1.413 ± 0.202 (A)	0.223 ± 0.065 (A)
Kayanne			
Pot	0.468 ± 0.06 (A)	2.980 ± 0.193 (A)	0.125 ± 0.029 (A)
RhizoTube	0.422 ± 0.03 (A)	2.599 ± 0.292 (B)	0.118 ± 0.025 (A)

Biomass, shoot over root biomass ratio, nodule over nodulated root biomass ratio and mean nodule weight of two pea genotypes (Cameor, Kayane) cultivated either in RhizoTubes or pots were measured after 18 days since sowing. Different capital letters in parentheses indicate significant differences (P < 0.05) between traits measured in RhizoTube or in pot

**Fig. 5:**
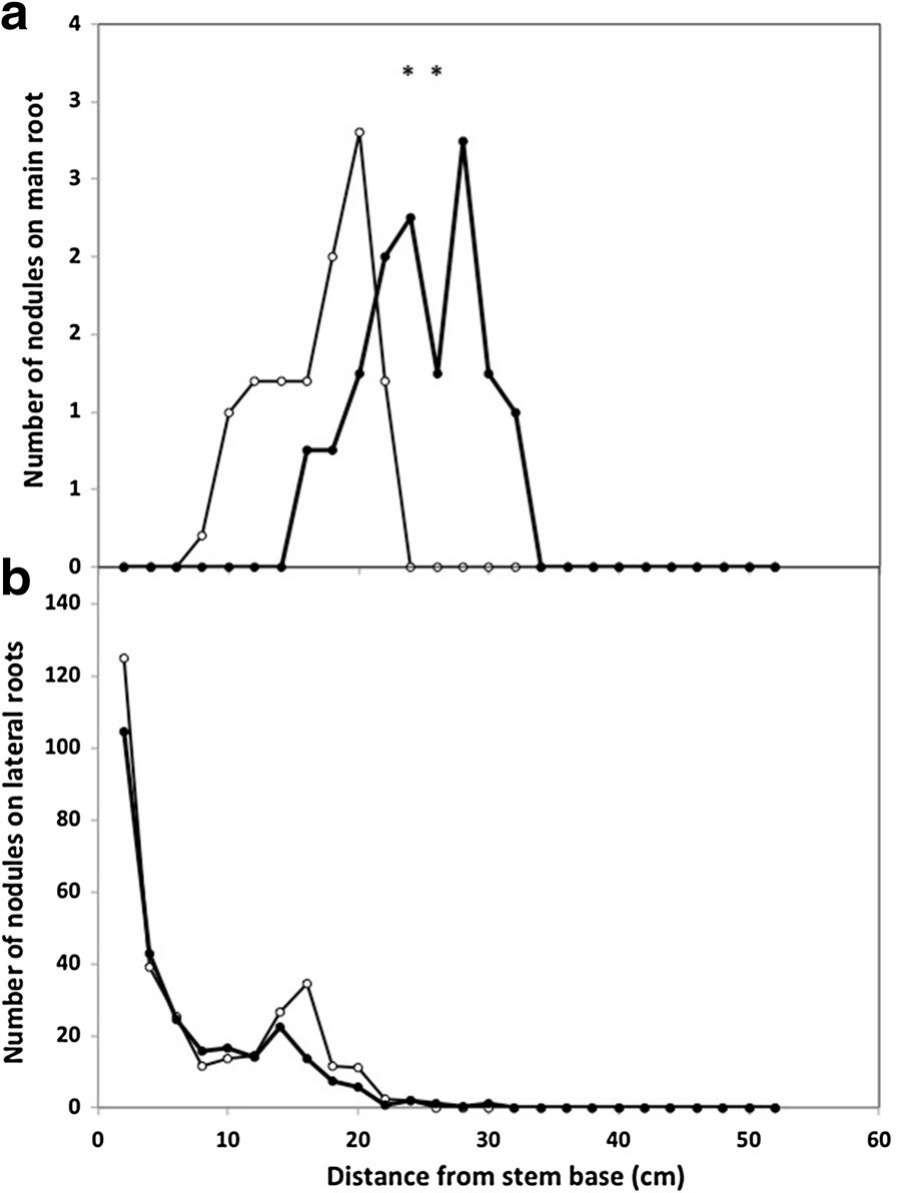
Nodule distribution on main root (**a**) and number of nodules on lateral roots (**b**) measured on roots of plants of *Pisum sativum* (Kayanne genotype) grown for 28 days without soil mineral nitrogen either in pots (*open circle*) or in RhizoTubes (*closed circles*). Data are given as the mean ± SE (n = 4). *Asterisks* indicate significant differences between traits measured in RhizoTube or in pot for P < 0.05

A deeper analysis of nodule distribution along the main and lateral roots was conducted on a set of Kayanne plants cultivated for 28 days without soil mineral nitrogen. While the number of nodules counted on lateral roots was very similar between containers (Fig. 5b), their distribution along the main root differed (Fig. 5a). In both RhizoTubes and pots there was an upper main root zone without any nodules, followed downward by a 16–20 cm long root zone bearing 1–3 nodules per cm, and lastly the terminal region without nodules. However, the main nodule-containing root zone was around 6 cm higher in RhizoTubes compared to pots (Fig. 5a).

### Mineral N response of contrasted plant species in RhizoTubes and pots

Besides legumes, whose root architecture and compartments (i.e. nodules and roots) are highly flexible, other plant species have contrasting responses to soil N variation. In experiment 4, the response of plant growth to mineral N was assessed for a poorly and a highly nitrophilic species, rapeseed and vulpia, respectively, either in RhizoTubes or in pots. The nitrogen response of these plant species was determined by changes in root dry matter and plant leaf area in our two growing containers (Table 4).

**Table 4:** Response of two weed species cultivated either in RhizoTubes or pots

	Response of root dry matter to soil-nitrogen	Response of leaf area to soil-nitrogen
Rapeseed		
Pot	3.48 ± 0.87 (A)	3.95 ± 0.56 (A)
RhizoTube	2.47 ± 0.39 (A)	2.21 ± 0.31 (B)
Vulpia		
Pot	1.08 ± 0.28 (A)	2.33 ± 0.36 (A)
RhizoTube	1.08 ± 0.26 (A)	1.42 ± 0.36 (B)

Response of root biomass and plant leaf area to soil-nitrogen for two weed species (rapeseed and Vulpia) cultivated either in RhizoTubes or pots was assessed by the ratio of the trait value at high soil-nitrogen to the trait value at low soil-nitrogen (mean value ± S.E., n = 5). Trait values were measured 51 days after sowing. Plant leaves and roots were separated and their biomasses were measured as in “Methods”. Leaf area was measured with a leaf area meter. Different capital letters in parentheses indicate significant differences between traits measured in RhizoTube or in pot for P < 0.05 (*)

**Fig. 6:**
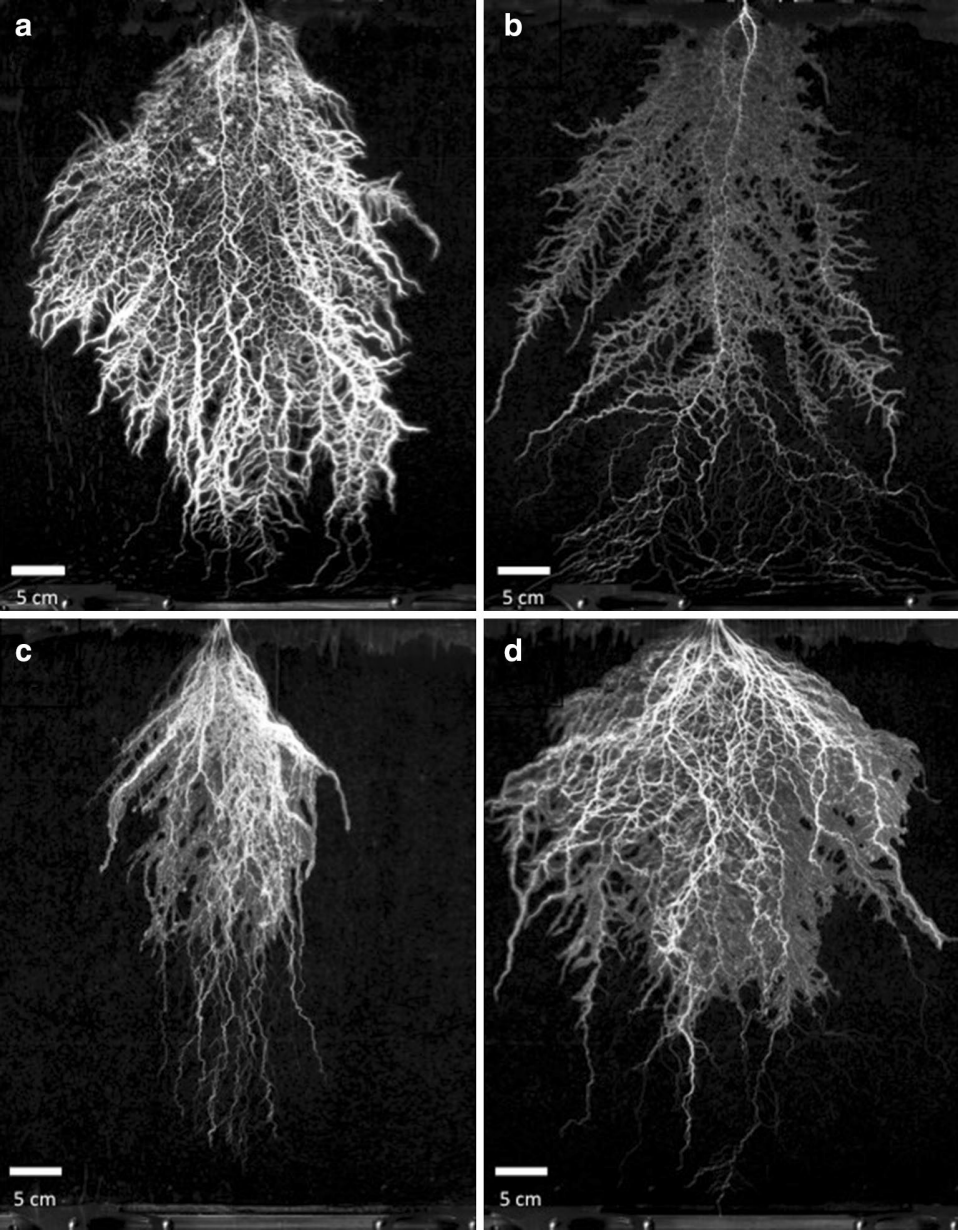
Root system of **a** rapeseed at high soil-nitrogen, **b** rapeseed at low soil-nitrogen, **c** Vulpia at high soil-nitrogen and **d** Vulpia at high low-nitrogen. Images were taken at 29 (**a**, **b**) and 51 days (**c**, **d**) after sowing for rapeseed and Vulpia respectively

Growing those species in RhizoTubes permitted the acquisition of root system images using the RhizoCab (Fig. 6). However both species developed very thin and entangled roots, which precluded detailed manual RSA measurements. Root biomass analysis demonstrated that the response to soil nitrogen was significantly higher for oilseed rape than for Vulpia but not significantly different between container types. Root dry matter and leaf area responses to soil mineral nitrogen were highest for the nitrophilic species (Table 4). Although the change in leaf area was significantly less in rhizotubes than in pots for both species, the leaf area of oilseed rape was more responsive than that of Vulpia to mineral N both in rhizotubes (P = 0.017) and in pots (P = 0.001).

### Water stress responses of two plant species in RhizoTubes and pots

In order to evaluate plant responses to water stress in RhizoTubes and in pots, a drought period was applied in experiment 5 to two different legume species (*pea* and *Medicago*) both in pots and RhizoTubes. Soil water retention of approximately 100 and 40 % of full capacity were applied for well-watered (WW) and water deficit (WS) treatments respectively. The effect of water restriction on shoot, root and nodule dry matter was compared for WW and WS plants (Table 5). Results were expressed as the ratio of dry matter under water deficit to dry matter of well-watered plants.

**Table 5:** Water deficit modulation of pea and *Medicago truncatula* compartments in pots or RhizoTubes

	Shoots	Roots	Nodules
*Pisum sativum*			
Pots	−19.9 ± 9.8 (A)	1.6 ± 14.4 (A)	−41.2 ± 11.9 (A)
RhizoTube	−32.6 ± 13.9 (B)	−7.4 ± 14.2 (B)	−36.9 ± 12.8 (A)
*Medicago*			
Pots	−26.5 ± 30.0 (A)	−11.5 ± 34.5 (A)	−15.9 ± 46.1 (A)
RhizoTube	−32.4 ± 20.5 (A)	−26.1 ± 23.4 (A)	−27.9 ± 23.3 (A)

Modulation of biomass allocation to shoot, roots and nodules of pea (Kayanne genotype) and Medicago truncatula plants subjected to a water stress versus well-irrigated plants was measured as the ratio of the difference in biomass of shoots (BMS), roots (BMR) or nodules (BMN) between water stress (WS) and well watered (WW) plants to the biomass of well watered plants (n = 5). As an example for shoots, (BMS_WS_ − BMS_WW_) × 100/BMS_WW_

Whatever the container or species, water deficit impacted less root biomass than either shoot or nodule biomass (Table 5). The water deficit effect was however less severe on pea roots than on Medicago roots but the reverse was observed for plant nodules. Shoot and root growth were slightly more depressed in pea grown in RhizoTubes than in pots. The response of nodule biomass to water deprivation was similar for both species whether cultivated in pots or RhizoTubes (Table 5).

### Root colonization by *P. fluorescens* C7R12

In order to check if microbial strains could persist in RhizoTubes, the bacterial strain *Pseudomonas fluorescens* C7R12 was inoculated after sowing pea and wheat plants. The goals of this assay were to: (1) characterize the bacterial cells concentrations on plant roots after 4 weeks of culture (measure of bacterial colonies on KING B agar plates) and (2) test if the method used to disinfect RhizoTubes (sodium hypochlorite solution) was effective enough to eliminate *P. fluorescens* cells after harvesting. This last point was performed in order to avoid microbial contamination for the following cultures.

*Pseudomonas fluorescens* C7R12 quantification on KING B agar plates showed a good persistence of the bacterial strain on roots in RhizoTubes. After 4 weeks of culture, the LOG of the densities of *P. fluorescens* C7R12 on roots were 6.19 ± 0.26, 5.69 ± 0.47 and 5.77 ± 0.29 CFU/g for respectively wheat, pea and wheat and pea grown together. Moreover, after culturing, when RhizoTubes were disinfected in sodium hypochlorite solution and checked for *P. fluorescens* C7R12 persistence, no bacterial colonies were detected on agar plates after cultures, indicating that the bacterial strain did not persist in the RhizoTubes after disinfection.

## Discussion

Ideally, pre requisites for root phenotyping are to visualize the whole root system at high resolution, and to identify possible changes in root system events, resulting from variations in biotic and abiotic environmental conditions, between genotypes or species. The system should allow root trait phenotyping dynamically and non-invasively. It should also accommodate a sufficient number of biological units to gain statistical power and also at high throughput because current applications of quantitative genetics require trait measurement of hundreds of genotypes (i.e. thousands of plants). Lastly, this should require minimal manual/human effort.

Root growth and architecture can be modulated by the rooting medium in which roots are grown. Root growth has to face physical constraints in soil while there are none in hydroponics. In pots these depend upon the root volume and pot size [54]. In RhizoTubes physical constraints force the root to grow in 2D and it is hence important to evaluate how this modifies plant responses to environmental abiotic and biotic factors as compared to growth in pots. Root growth and architecture are not only modulated by the availability of soil resources but also by soil microorganisms [55].

### RhizoTubes are suitable for plant growth; root architectural traits of plants growing in RhizoTubes or pots are similar

RhizoTubes allowed similar plant growth to that observed in pots, for all the plant species studied. Thus, RhizoTubes are suitable tools for studying the root systems of various species. Coupled with a high-definition camera, movable in 2D, we had high-resolution access to the root traits such as root and nodule diameters, emerging nodules and hyphae.

In our study, pea plants allocated similar amounts of their biomass to roots in RhizoTubes and in pots (Table 2). However, pea main root length was longer in RhizoTubes as previously reported for growth in hydroponics (or growth pouches) versus pots [22]. Presumably these results from the lower mechanical resistance in RhizoTubes compared to pots where roots encounter substrate particles. The depth of the RhizoTube (50 cm) is attained after around 1.5 month’s growth, depending on species and growing conditions. As such, root phenotyping during the first month of growth generally allows undisturbed root growth and architecture with easy visualization of individual roots. Later on, roots generally start to entangle and root architecture starts to be constrained by reaching the bottom of the RhizoTube. As such, the deeper environment resulting from growth in RhizoTube might explain the higher length of main root that was observed (Table 2). Similarly a shallower and plateauing distribution profile of laterals on main roots and then fewer, smaller, lateral roots were observed at the bottom of RhizoTubes (Fig. 4). In accordance with the above hypothesis, the number of secondary lateral roots in pots displayed a sharp peak at around 20 cm from the main root base, a distance which corresponds exactly to the pot depth (Fig. 4c, d).

### RhizoTubes show similar phenotypic variations between genotypes than those observed in pots

In absence of mineral nitrogen, legumes carry out endosymbiotic nitrogen fixation with rhizobial bacteria, within a specialized structure known as the nodule, which increases the complexity of root system analyses. Nodule biomass and number determine plant nitrogen status [56, 57] and constitute key phenotypic traits of nodulated legume roots.

Similar nodule distribution was observed for pea plants cultivated in RhizoTubes or pots, especially for nodules borne by laterals, which are by far the more numerous. Comparing genotypes, a similar plant biomass and similar biomass partitioning between shoots and roots were observed for Cameor and Kayanne genotypes grown either in pots or in RhizoTubes (Table 3).

RhizoTubes allowed us to rank both oilseed rape and *Vulpia* species as a function of the response of their root biomasses to soil nitrogen with a ranking similar to that in pots. This demonstrates that RhizoTubes allow not only to compare plant genotype/species in similar environmental conditions but also to compare their ranking in their responses to environmental factors. Although the response of plant leaf area to soil mineral N observed in RhizoTubes was lower than that observed in pots, the ranking of oilseed rape and *Vulpia* plant species appeared similar. RhizoTubes were suitable tools for highlighting the more nitrophilic response of oilseed rape as compared to *Vulpia* already demonstrated by changes in leaf area [58].

### RhizoTubes are suitable for studying water stress effects on plants, which exhibit similar root responses to those grown in pots

RhizoTubes allowed us to study shoot, root and nodule growth responses to drought in the same ranges as those observed in pots. This applied both to plants growing in a large volume of soil in pots and in the 2D environmental conditions of RhizoTubes without direct contact of roots with soil. Nodule biomass allocation was repressed for both pea and Medicago under water stress conditions as compared to well-watered conditions (Table 4). Whereas drought impact on biomass accumulation was similar for Medicago in RhizoTubes as compared to pots, pea shoot and root biomasses were more reduced when grown in RhizoTubes. This may arise from the difficulty of applying similar water deficit regimes in pots and RhizoTubes. Although we tried to optimize irrigation for plants in RhizoTubes (through sequential addition of nutrient solutions) and in pots (nutrient solution supplies being gravimetricaly controlled), possibly water supplies of plants in RhizoTubes and in pots did not match perfectly so that to lead to a similar level of water stress sensed by plants. This highlights the need to accurately adjust nutrient solution supplies in frequency and amount among containers to get similar plant responses to drought. Despite the tuning required, RhizoTubes are well suited for drought stress response studies on legume plants.

### RhizoTubes are suitable systems for characterizing plant–microorganism interactions

Legume plants grown with ample nitrogen supply in the rooting medium do not establish nodulation with rhizobia [14, 57, 59], as consistently observed here both in pots and RhizoTubes. However in absence of soil mineral nitrogen, as demonstrated above, RhizoTubes allow legume-rhizobia symbiosis to be established, as in pots. This is made possible as rhizobia cross the membrane, which in turn also demonstrates that early plant–bacteria communication operates successfully via rhizodeposit and nod factor exchanges through the RhizoTube membrane between plant and bacteria. The very slight shift of nodule distribution profile towards the lower part of the main root in RhizoTubes might be explained either by mechanical constraints resulting from the lower root volume and higher substrate resistance of soil in pots than in the RhizoTube.

The production of mycorrhiza-colonized grapevine plants was possible in RhizoTubes with a total mycorrhization level and a root age-dependency similar to that encountered in pots. These observations are in accordance with previous results obtained for grapevines grown in pots, with the same inoculum, showing the reliability of the RhizoTube for mycorrhiza studies (unpublished data). Interestingly, the visualization of the mycelium was possible at 3600 ppi (Fig. 3), illustrating the high resolution of imaging, even if image-based quantification was not possible (as the mycelium is in part hidden by roots).

In microbial ecology, assessing the impact of microbial strains, populations or communities on root development remains a challenge. The strategy usually applied consists of inoculating plants with different microbial strains and then collecting, analyzing and comparing root architectures at different developmental stages [60, 61]. However, these experiments are not easy to manage in term of numbers of replicates for each treatment and sampling date and are usually time consuming. In order to determine whether free-living microorganisms could persist in RhizoTubes, the bacterial strain *P. fluorescens* C7R12 was inoculated on pea and wheat roots. The bacterial strain persisted in the root systems of plants in RhizoTubes, with densities of *P. fluorescens* C7R12 comparable to that observed in other systems [60]. Moreover, the strain was completely removed after sodium hypochlorite disinfection, allowing the subsequent re-use of RhizoTubes for plant–microbe studies. Taken together, these results showed that RhizoTubes are suitable for inoculating both symbiotic (rhizobia and arbuscular mycorrhizal fungi) and free-living microorganisms on plant roots. RhizoTubes can therefore be used to measure kinetically and non-invasively the interactions between plant roots and microorganisms.

### RhizoTube and RhizoCab pros and cons

Growing plants in RhizoTubes did not change the extent of the plant’s response to the environmental treatments tested here. The suite of tools presented here is powerful not only for measuring phenotypic traits but also for ranking genotypes for a given response to environmental factors.

The study of RSA traits such as lateral root number and length is complicated by the inaccessibility of the soil matrix. RhizoTubes allow visual access to the entire root system. Another advantage is that the cylindrical configuration yields a surface area of about 0.5 m^2^, which for flat rhizotrons would be problematic during movement of plants on conveyors to imaging cabins.

Our system is also highly flexible as it allows working with a range of substrates, because plant roots never enter in direct contact with the substrate. However thanks to the RhizoTube membrane, the substrate can still have its role of hosting microbes which can receive their share of rhizodeposits while at the same time plants receive their nutrient solution and establish symbioses with introduced bacteria or fungi. RhizoTubes are however not compatible with all substrates. The substrates used in RhizoTubes should be dense enough to exert a uniform pressure on the membrane so that roots grow tightly between the membrane and the transparent outer tube. This allows creating a mechanical constraint on roots, which can be modulated when substrate is filled in the RhizoTube. Substrates such as peat do not allow sufficient pressure on membrane, due to their density and cannot be used in RhizoTubes. In order to avoid substrate particles masking roots and so perturbing image analysis, the RhizoTube substrate has not to cross the membrane, which has so been designed with a low porosity. However, the membrane porosity cannot be lowered too much, as it would be detrimental to water and nutrient exchanges with the plant roots. As such, filling RhizoTubes with natural soil only is not recommended. However, in case of studies concerning between plant and soil microbial community interactions, natural soil hosting these microbial communities can be mixed with sand. Substrates such as sand, perlite/sand, sable/billes d’argile, atapulgite/billes d’argile mixtures have been successfully used. These allow water and nutrient solution supply management, for a given water retention capacity. In addition there is no need for tedious root separation from the substrate during harvest and even the thinnest roots are recovered. The membrane allows having soil-free root material, a disadvantage when plants are cultivated in pots. Lastly, while inevitably variable amounts of root parts, depending on both species and their age, get lost during the root harvest and washing process in pots, RhizoTubes in contrast allows the recovery of the entire roots giving unbiased plant root biomass comparison during the various experiments.

One of the major difficulties when plants grow deep in pots or in RhizoTubes is overlapping roots, which renders individual root identification tedious in branched or old root systems. Although analyzing root systems of mature plants in our systems remains a bottleneck, a reasonably long experimental duration can be assured for a given species in our system, which avoids (or reduces) the need for untangling roots harvested from soil pots to organize them into a 2D conformation, and precludes root damage.

Our cultivation system is flexible enough to allow growing plants from various species at several developmental stages (Table 6). The time of cultivation required for at least one plant root to reach the bottom of the RhizoTube was recorded for a variety of plant species (Table 6). It varied according to the plant root architecture, the root systems from species such as wheat reaching the RhizoTube bottom more rapidly than others having a more superficially developed root.

**Table 6:** Duration of experiment and developmental stages reached before at least one root of various plant species reach the bottom of RhizoTube

Species	Number of days since sowing before at least one root reaches the bottom of the RhizoTube	Sum of degree days (base 0) since germination	Developmental stage
*Trigonella foenum*-*graecum*	42 ± 3.64	888	6.1
*Vicia faba*	46 ± 3.02	978	10.9
*Phaseolus vulgaris* L.	43 ± 1.41	911	7.0
*Lens culinari*	36 ± 4.63	755	14.1
*Lupinus Albus* L.	30 ± 2.26	639	8.6
*Hordeum vulgare*	22 ± 0.5	488	2.8
*Brassica napus* L.	30 ± 2.5	623	8.5
*Pisum Sativum* L.	35 ± 3.15	755	11.7
*Cicer arietinum*	32 ± 3.18	685	14.2
*Glycine max*	33 ± 3.81	700	6.3
*Vicia sativa*	35 ± 4.48	751	16.8
*Vicia narbonensis* L.	37 ± 5.61	794	8.9

Plant species were sown and cultivated in RhizoTubes using optimum nutrient solution to evaluate the time (in days or degree days) and developmental stage when at least one root reached the RhizoTube bottom. Value are expressed as mean value ± S.E., n = 5

However, RhizoTubes can be used for an extended period of time beyond 10–20 days depending on the speed of root development which is variable both for a given species according to environmental conditions and between plant species. This limitation mostly arises from the RhizoTube dimensions that will be increased in future development up to 1 m height and 0.35 m diameter, for other platforms having specific needs of either increased duration of plant root observation or devoted to work with species having much larger root systems. Their use implies that the platform’s specifications can handle them, with sufficient phenotyping cabin height (i.e. higher than the 1.6 m of our 4PMI phenotyping cabins) for plant entry, sufficient depth of view for zenithal images and powerfull motors to tackle the higher RhizoTubes weight.

**Fig. 7:**
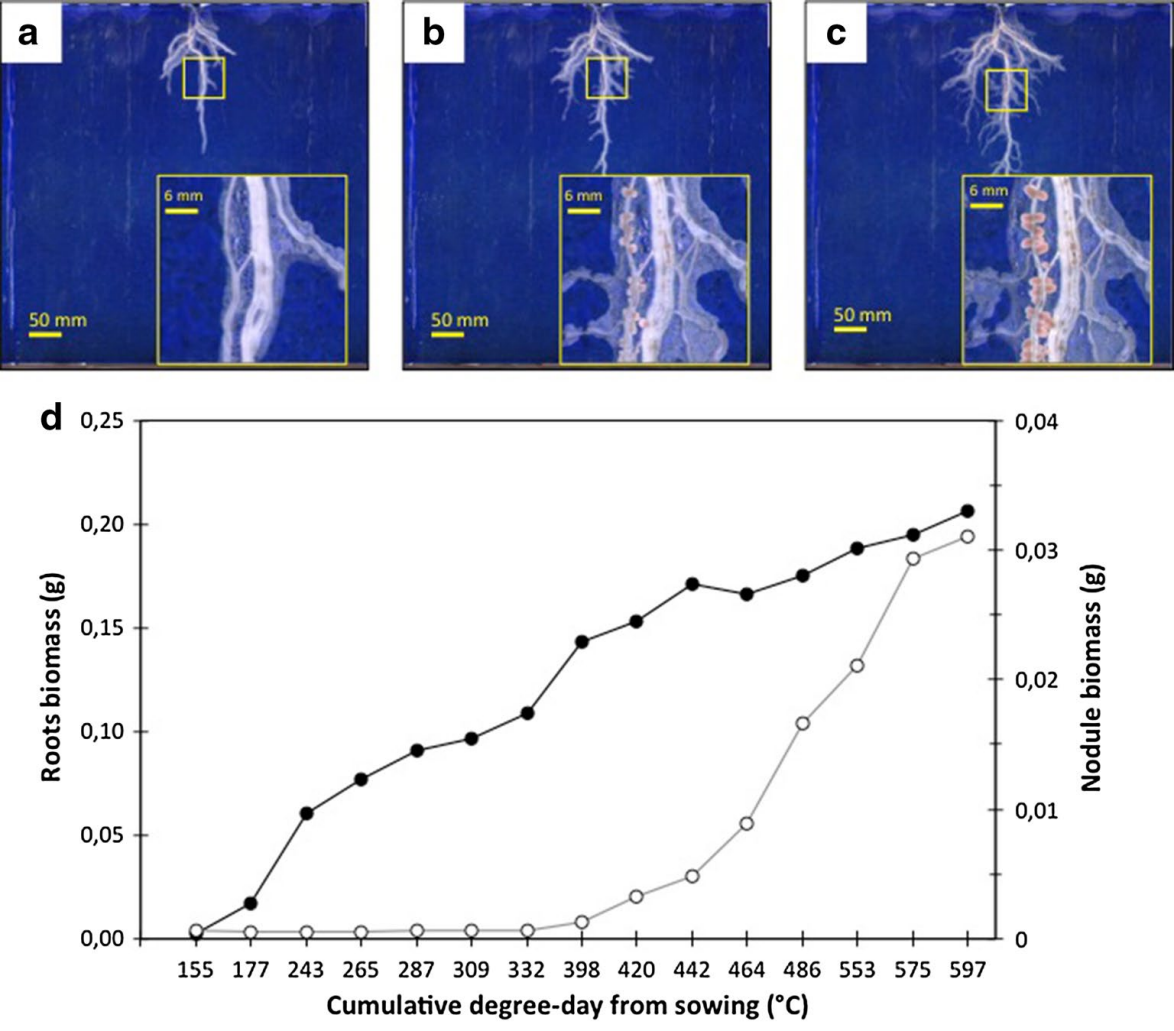
Root and nodule growth dynamic obtained by digital imaging analysis. Images of pea plant (kayanne genotype) grown in RhizoTube without soil mineral nitrogen in experiment 3 were realized using RhizoCab at resolution of 600 ppi and RVB light. Examples of nodulated roots images (with a focus in the *yellow colored square*) taken at 309, 464 and 597 degree-days are shown in **a**, **b** and **c** respectively. Image segmentation allows to obtain the pixels numbers for roots and nodules. These values are then converted in biomass (grams) using a calibration curves (data not shown). As such, nodule biomass (*open circles*) and root biomass (*closed circles*) can be dynamically estimated during the growth cycle (**d**)

Our equipment allow to dynamically following growth processes (root elongation, increase in nodule biomass, nodulation waves etc.) of a given plant throughout a large span of its growth cycle (an example is given in Fig. 7 for root and nodule growth dynamic). This decreases the inter-plant heterogeneity resulting from destructive observations on different plant samples across time. It also reduces the number of replicates and the overall cost of experiments.

Lastly, RhizoTubes can be moved on conveyors thanks to their specially designed base. This allows their implementation in high throughput platforms using the “plant to sensor” concept, comprising automatic watering and imaging, completing the shoot imaging usually deployed on such platforms.

### Image quality and analysis

The resolution of the images (from 42 µm per pixel at 600 ppi down to 7 µm per pixel at 3600 ppi) allows detection of the thinnest roots and nodules (Fig. 2c, e) and to see hyphae (Fig. 3). Image acquisition is very easy and fast in our system but analysing them may be rate limiting. In this study root phenotypic traits were manually determined. However powerful algorithms under development will allow an automatic extraction of a variety of nodule and root morphometric traits from the images obtained with RhizoCab.

Digital imaging used in 4PMI for shoot and root phenotyping allows extracting from individual images non-invasively and throughout a large spanning of plant lifecycle a number of phenotypic traits. Image acquisition, the first step in a high-throughput phenotyping work flow, is easily performed using RhizoTubes and the associated imaging cabin RhizoCabs for root traits while shoot images are acquired sequentially for both plants growing in pots or in RhizoTubes in the other imaging cabins of 4PMI. Every shoot measurement made on a pot, can be ported on a RhizoTube because it can be simply considered as a 50 cm high pot. Subsequently, a binary image of the RGB original image or a single wavelength is used to improve image segmentation. Both plant shoot support frames and RhizoTube membranes are colored in blue. This greatly facilitates image processing and segmentation. Focusing here on root images, while a number of softwares can already be used to extract phenotypic traits (http://www.plant-image-analysis.org/), specialized image analysis software is still necessary for producing an automatic, rapid extraction of root phenotypic traits of interest for the variety of plant root systems that may be encountered in research [41]. Towards this challenge, image analysis algorithms are under development in our group in collaboration with scientists possessing the necessary programming skills [62]. With this dedicated software (Han S., unpublished) numerous morphological parameters will be automatically and dynamically extracted and quantified from root images such as projected area of root and nodules, number, length, diameters and positions of the various roots and nodules on roots.

## Conclusions

The data presented demonstrate that growing plants in RhizoTubes overall yields a fair representation of what is obtained in pots under optimal, drought and variable nitrogen nutrition conditions for the species used. Whereas growth in pots or in RhizoTubes does not perfectly mimic field conditions and indeed no artificial systems can perfectly simulate natural conditions, RhizoTubes can be used to test environmental or genetic variations in a given growth condition. Our system will provide the scientific community with up-to-date and very innovative tools for accessing high resolution plant root traits. This will allow analyses of key phenotypic traits for root architecture and growth, comparable to those obtained either in controlled conditions or in the field.

The scientific community has recently made a huge effort in assembling their expertise of complementary phenotyping tools and methods either in the context of French (i.e. Phenome, French Plant Phenotyping Network, https://www.phenome-fppn.fr/), English (UK Plant Phenotyping Network, http://www.ukppn.org.uk/), German (Deutch Plant Phenotyping Network, www.dppn.de/), European (European Plant Phenotyping Network, http://www.plant-phenotyping-network.eu/), International networks (International Plant Phenotyping Network, IPPN, www.plant-phenotyping.org/). The number and rate of developments in automated phenotyping are impressive, and now a broad range of root phenotyping platforms is available. Our root phenotyping platform does not claim to produce universal standards. The 4PMI platform and its root phenotyping equipment have been tested and validated for a variety of plants and environmental conditions. However, in this paper we underline that its domain of applicability is restricted by both phenological stages and growth of plants, and the variety of applied stresses. While we show it is possible to inoculate microorganisms in RhizoTubes where they persist and colonize plant roots, further experiments are needed to characterize the impact of soil microorganisms on the different root traits in this system.

These tools and methods will advance knowledge of the processes involved in root development to provide a basis for development of crops that can better manage the effects of limited water or nutrient supply. This will allow functional validation of the roles of genes. The platform will be invaluable for strengthening quantitative and association genetic studies for root traits in order to decipher the molecular basis for the adaptation of structural/functional traits to (a)biotic stresses. Thanks to modelling, generic relations between phenotypic traits and genes will be characterized.

## Abbreviations

RSA: root system architecture
RFID: Radio Frequency Identification
2D: two dimension
D: three dimensions
LED: light-emitting diode
RGB: red, green and blue
MB: megabyte
BMP: bitmap
PNG: portable network graphics
CFU: Colony Forming Unit
PPI: pixel per inch

## References

[1] Tilman D, Balzer C, Hill J, Befort BL (2011). Global food demand and the sustainable intensification of agriculture. PNAS.

[2] Lynch JP, Brown KM (2012). New roots for agriculture: exploiting the root phenome. Philos Trans R Soc B Biol Sci.

[3] Fiorani F, Schurr U (2013). Future scenarios for plant phenotyping. Ann Rev Plant Biol.

[4] Cabrera-Bosquet L, Fournier C, Brichet N, Welcker C, Suard B, Tardieu F (2016). High throughput estimation of incident light, light interception and radiation-use efficiency of thousands of plants in a phenotyping platform. New Phytol.

[5] Coupel-Ledru A, Lebon É, Christophe A, Doligez A, Cabrera-Bosquet L, Péchier P, Hamard P, This P, Simonneau T (2014). Genetic variation in a grapevine progeny (*Vitis vinifera* L. cvs Grenache × Syrah) reveals inconsistencies between maintenance of daytime leaf water potential and response of transpiration rate under drought. J Exp Bot.

[6] Golzarian MR, Frick RA, Rajendran K, Berger B, Roy S, Tester M, Lun DS (2011). Accurate inference of shoot biomass from high-throughput images of cereal plants. Plant Methods..

[7] Tuberosa R, Sanguinetti MC, Landi P, Giuliani MM, Salvi S, Conti S (2002). Identification of QTLs for root characteristics in maize grown in hydroponics and analysis of their overlap with QTLs for grain yield in the field at two water regimes. Plant Mol Biol.

[8] Wasson AP, Richards RA, Chatrath R, Misra SC, Prasad SS, Rebetzke GJ, Kirkegaard JA, Christopher J, Watt M (2012). Traits and selection strategies to improve root systems and water uptake in water-limited wheat crops. J Exp Bot.

[9] Nagel KA, Kastenholz B, Jahnke S (2009). Temperature responses of roots: impact on growth, root system architecture and implications for phenotyping. Funct Plant Biol.

[10] Bengough AG, McKenzie BM, Hallett PD, Valentine TA (2011). Root elongation, water stress, and mechanical impedance: a review of limiting stresses and beneficial root tip traits. J Exp Bot.

[11] Pfeifer J, Faget M, Walter A, Blossfeld S, Fiorani F, Schurr U, Nagel KA (2014). Spring barley shows dynamic compensatory root and shoot growth responses when exposed to localised soil compaction and fertilisation. Funct Plant Biol.

[12] Acuña TB, Wade LJ (2012). Genotype × environment interactions for root depth of wheat. Field Crops Res..

[13] Cairns JE, Impa SM, O’Toole JC, Jagadish SVK, Price AH (2011). Influence of the soil physical environment on rice (*Oryza sativa* L.) response to drought stress and its implications for drought research. Field Crops Res.

[14] Voisin A-S, Salon C, Munier-Jolain NG, Ney B (2002). Effect of mineral nitrogen on nitrogen nutrition and biomass partitioning between the shoot and roots of pea (*Pisum sativum* L.). Plant Soil.

[15] López-Bucio J, Cruz-Ramírez A, Herrera-Estrella L (2003). The role of nutrient availability in regulating root architecture. Curr Opin Plant Biol..

[16] Lambers H, Mougel C, Jaillard B, Hinsinger P (2009). Plant–microbe–soil interactions in the rhizosphere: an evolutionary perspective. Plant Soil.

[17] Trachsel S, Kaeppler SM, Brown KM, Lynch JP (2011). Shovelomics: high throughput phenotyping of maize (*Zea mays* L.) root architecture in the field. Plant Soil.

[18] Vocanson A, Roger-Estrade J, Boizard H, Jeuffroy MH (2006). Effects of soil structure on pea (*Pisum sativum* L.) root development according to sowing date and cultivar. Plant Soil.

[19] Bourion V, Laguerre G, Depret G, Voisin AS, Salon C, Duc G (2007). Genetic variability in nodulation and root growth affects nitrogen fixation and accumulation in pea. Ann Bot.

[20] Clark RT, Famoso AN, Zhao KY, Shaff JE, Craft EJ, Bustamante CD, McCouch SR, Aneshansley DJ, Kochian LV (2013). High-throughput two-dimensional root system phenotyping platform facilitates genetic analysis of root growth and development. Plant Cell Environ.

[21] Voisin AS, Cazenave AB, Duc G, Salon C (2013). Pea nodule gradients explain C nutrition and depressed growth phenotype of hypernodulating mutants. Agron Sust Dev.

[22] Clark RT, MacCurdy RB, Jung JK, Shaff JE, McCouch SR, Aneshansley DJ, Kochian LV (2011). Three-dimensional root phenotyping with a novel imaging and software platform. Plant Phys..

[23] Hargreaves CE, Gregory PJ, Bengough AG (2009). Measuring root traits in barley (*Hordeum vulgare* ssp. vulgare and ssp*. spontaneum*) seedlings using gel chambers, soil sacs and X-ray microtomography. Plant Soil.

[24] Nagel KA, Putz A, Gilmer F (2012). GROWSCREEN-Rhizo is a novel phenotyping robot enabling simultaneous measurements of root and shoot growth for plants grown in soil-filled rhizotrons. Funct Plant Biol.

[25] Slovak R, Göschl C, Su X, Shimotani K, Shiina T, Busch W (2014). A scalable open-source pipeline for large-scale root phenotyping of Arabidopsis. Plant Cell.

[26] Bourion V, Rizvi SMH, Fournier S, de Larambergue H, Galmiche F, Marget P, Duc G, Burstin J (2010). Genetic dissection of nitrogen nutrition in pea through a QTL approach of root, nodule, and shoot variability. Theor Appl Gen.

[27] Pages L (1992). Mini-rhizotrons transparents pour l’étude du système racinaire de jeunes plantes. Application à la caractérisation du développement racinaire de jeunes chênes (*Quercus robur*). Can J Bot.

[28] Ytting NK, Andersen SB, Thorup-Kristensen K (2014). Using tube rhizotrons to measure variation in depth penetration rate among modern North-European winter wheat (*Triticum aestivum* L.) genotypes. Euphytica.

[29] Topp CN, Iyer-Pascuzzi AS, Anderson JT (2013). 3D phenotyping and quantitative trait locus mapping identify core regions of the rice genome controlling root architecture. PNAS.

[30] Fang S, Clark R, Liao H. 3D quantification of plant root architecture in situ. In: Stefano M, editor. Measuring roots. Heidelberg: Springer; 2012. p. 135–148.

[31] Hund A, Trachsel S, Stamp P (2009). Growth of axile and lateral roots of maize: I development of a phenotying platform. Plant Soil.

[32] Adu MO, Chatot A, Wiesel L, Bennett MJ, Broadley MR, White PJ, Dupuy LX (2014). A scanner system for high-resolution quantification of variation in root growth dynamics of Brassica. J Exp Bot.

[33] Le Marié C, Kirchgessner N, Marschall D, Walter A, Hund A (2014). Rhizoslides: Paperbased growth system for non-destructive, high throughput phenotyping of root development by means of image analysis. Plant Methods.

[34] Fang SQ, Yan XL, Liao H (2009). 3D reconstruction and dynamic modeling of root architecture in situ and its application to crop phosphorus research. Plant J.

[35] Ribeiro KM, Barreto B, Pasqual M, White PJ, Braga RA, Dupuy LX (2014). Continuous, high-resolution biospeckle imaging reveals a discrete zone of activity at the root apex that responds to contact with obstacles. Ann Bot.

[36] Downie H, Holden N, Otten W, Spiers AJ, Valentine TA, Dupuy LX (2012). Transparent soil for imaging the rhizosphere. PLoS One.

[37] Bucksch A, Burridge J, York LM, Das A, Nord E, Weitz JS, Lynch JP (2014). Image-based high-throughput field phenotyping of crop roots. Plant Phys.

[38] Ali-Khan ST, Snoad B (1977). Root and shoot development in peas. I. Variability in seven root and shoot characters of seedlings. Ann Appl Biol.

[39] McPhee K (2005). Variation for seedling root architecture in the core collection of pea germplasm. Crop Sci.

[40] Thorup-Kristensen K (1998). Root growth of green pea (*Pisum sativum* L.) genotypes. Crop Sci.

[41] Atkinson JA, Rasmussen A, Traini R, Voß U, Sturrock C, Mooney SJ (2014). Branching out in roots: uncovering form, function, and regulation. Plant Physiol.

[42] Bellini C, Pacurar DI, Perrone I (2014). Adventitious roots and lateral roots: similarities and differences. Annu Rev Plant Biol.

[43] Walter A, Spies H, Terjung S, Kusters R, Kirchgessner N, Schurr U (2002). Spatio-temporal dynamics of expansion growth in roots: automatic quantification of diurnal course and temperature response by digital image sequence processing. J Exp Bot.

[44] Shi L, Shi TX, Broadley MR, White PJ, Long Y, Meng JL, Xu FS, Hammond JP (2013). Highthroughput root phenotyping screens identify genetic loci associated with root architectural traitsin *Brassica napus* under contrasting phosphate availabilities. Ann Bot.

[45] Leitner D, Felderer B, Vontobel P, Schnepf A (2014). Recovering root system traits using image analysis exemplified by two-dimensional neutron radiography images of lupine. Plant Physiol.

[46] Moradi AB, Conesa HM, Robinson B, Lehmann E, Kuehne G, Kaestner A, Oswald S, Schulin R (2009). Neutron radiography as a tool for revealing root development in soil: capabilities and limitations. Plant Soil.

[47] Mooney SJ, Pridmore TP, Helliwell J, Bennett MJ (2012). Developing X-ray computed tomography to non-invasively image 3-D root systems architecture in soil. Plant Soil.

[48] Pfeifer J, Kirchgessner N, Colombi T, Walter A (2015). Rapid phenotyping of crop root systems in undisturbed field soils using X-ray computed tomography. Plant Methods.

[49] Rascher U, Blossfeld S, Fiorani F (2011). Non-invasive approaches for phenotyping of enhanced performance traits in bean. Funct Plant Biol.

[50] van Dusschoten D, Metzner R, Kochs J, Postma JA, Pflugfelder D, Buehler J, Schurr U, Jahnke S (2016). Quantitative 3D analysis of plant roots growing in soil using magnetic resonance imaging. Plant Phys.

[51] Kim Khiook IL, Schneider C, Heloir MC, Bois B, Daire X, Adrian M, Trouvelot S (2013). Image analysis methods for assessment of H_2_O_2_ production and *Plasmopara viticola* development in grapevine leaves: application to the evaluation of resistance to downy mildew. J Microbiol Met.

[52] Lemanceau P, Alabouvette C (1991). Biological control of fusarium diseases by fluorescent pseudomonas and non-pathogenic fusarium. Crop Protection..

[53] Trouvelot A, Kough JL, Gianinazzi-Pearson V. Mesure du taux de mycorhization VA d’un système radiculaire. Recherche de méthodes d’estimation ayant une signification fonctionnelle. In: Gianinazzi-Pearson V, Gianinazzi S, editors. Mycorrhizae: physiology and genetics. Paris: INRA; 1986. p. 217–221.

[54] Poorter H, Bühler J, van Dusschoten D, Climent J, Postma JA (2012). Pot size matters: a meta-analysis of the effects of rooting volume on plant growth. Funct Plant Biol.

[55] Pivato B, Offre P, Marchelli S, Barbonaglia B, Mougel C, Lemanceau P, Berta G (2009). Bacterial effects on arbuscular mycorrhizal fungi and mycorrhiza development as influenced by the bacteria, fungi, and host plant. Mycorrhiza.

[56] Ruffel S, Freixes S, Balzergue S (2008). Systemic signaling of the plant nitrogen status triggers specific transcriptome responses depending on the nitrogen source in *Medicago truncatula*. Plant Phys.

[57] Jeudy C, Ruffel S, Freixes S, Tillard P, Santoni AL, Morel S, Journet EP, Duc G, Gojon A, Lepetit M, Salon C (2010). Adaptation of *Medicago truncatula* to nitrogen limitation is modulated via local and systemic nodule developmental responses. New Phytol.

[58] Moreau D, Milard G, Munier-Jolain N (2013). A plant nitrophily index based on plant leaf area response to soil nitrogen availability. Agron Sustain Dev.

[59] Moreau D, Voisin AS, Salon C, Munier-Jolain N (2011). The model symbiotic association between *Medicago truncatula* cv. Jemalong and Rhizobium meliloti strain, leads to N-stressed plants when symbiotic N_2_ fixation is the main N source for plant growth. J Exp Bot.

[60] Gamalero E, Trotta A, Massa N, Copetta A, Martinotti MG, Berta G (2004). Impact of two fluorescent pseudomonads and an arbuscular mycorrhizal fungus on tomato plant growth, root architecture and P acquisition. Mycorrhiza.

[61] Gamalero E, Lingua G, Tombolini R, Avidano L, Pivato B, Berta G (2005). Colonization of tomato root seedling by *Pseudomonas fluorescens* 92rkG5: spatio-temporal dynamics, localization, organization, viability and culturability. Microb Ecol.

[62] Han S, Cointault F, Salon C, Simon JC. Automatic detection of nodules in legumes by imagery in a phenotyping context. computer analysis of images and patterns. In: Azzopardi G, Petkov N, editors. Computer Analysis of Images and Patterns. Lecture notes in computer science. vol. 9257, Heidelberg: Springer; 2015. p. 134–145.

